# A Risk-Assessing Signature Based on Hypoxia- and Immune-Related Genes for Prognosis of Lung Adenocarcinoma Patients

**DOI:** 10.1155/2022/7165851

**Published:** 2022-09-28

**Authors:** Yue Wang, Jian Feng, Yang Liu, Tingyue Han

**Affiliations:** Cardio-Thoracic Surgery, Funing People's Hospital, Yancheng 224400, China

## Abstract

Lung Adenocarcinoma (LUAD) drastically influences human health. Tumor hypoxia and immunity impact hugely on the immunotherapeutic effect of LUAD patients. This study is aimed at exploring the prognostic markers associated with hypoxia and immunity in LUAD patients and evaluates their reliability. The relationship between hypoxia and immune-related genes and prognoses of LUAD patients was investigated by the univariate regression analysis. Gene Ontology (GO) and Kyoto Encyclopedia of Genes and Genomes (KEGG) methods were used to reveal the enriched pathways and biological processes of prognosis-related genes. Univariate, LASSO, and multivariate Cox regression analyses were used to construct a prognostic signature and verify its independence. The reliability of the signature was evaluated by the Principal Component Analysis (PCA), the Kaplan-Meier (K-M) curve, and the receiver operating characteristic (ROC) curve. Gene set enrichment analysis (GSEA), tumor mutational burden (TMB), and single-sample GSEA (ssGSEA) further verified the performance of the signature. Finally, a prognostic signature for LUAD was constructed based on 7 hypoxia- and immune-related genes. According to riskScores acquired from the signature, the test set was divided into groups, where the prognosis of high-risk patients was poor. The feature genes had good reliability, and the riskScore could be used as an independent prognostic factor for LUAD patients. Meanwhile, high TMB scores and low immune scores were found in high-risk patients, and feature genes were enriched in signaling pathways such as cell cycle and p53 signaling pathway. In sum, a prognostic signature based on 7 hypoxia- and immune-related genes was constructed.

## 1. Introduction

The morbidity and mortality of lung cancer (LC) are high worldwide. Statistic by International Agency for Research on Cancer (IARC) showed that 11.4% new cases of cancers are LC, only followed by breast cancer (11.7%) [1]. In addition, the mortality of lung cancer accounts for 18%, ranking the first globally [1]. Lung adenocarcinoma (LUAD) is the most frequent histological subtype of LC [2]. Progress has been made in LUAD-related research in the past decades, but the survival rate is not noticeably improved, with 70% of patients developing local progression or metastasis when they were first diagnosed [3–5]. In the above conditions, the impact of traditional interventions on patients is only revealed after treatment, which adds patients' pain. Hence, the clinician needs to take the treatment sensitivity and prognosis of patients into consideration for making further treatment plans.

Hypoxia is a typical feature of the tumor microenvironment. Cells were driven and became invasive in the hypoxia condition in hepatocellular carcinoma, colorectal cancer, and esophageal squamous cell carcinoma [6–8]. Evidence has shown that with hypoxia conditions, tumor cells can activate multiple transcription factors to further induce cell proliferation, invasion, and apoptosis [8, 9]. This enhances the drug resistance of tumor cells under a hypoxia environment, and hypoxia-induced cell function changes will affect the prognoses of patients [10]. Therefore, hypoxia is deemed to relate to drug resistance and poor treatment efficacy [11]. Research proved that a patient's prognosis can be predicted using hypoxia-related genes. Jiang et al. [12] confirmed that LBH is lowly expressed in glioma under hypoxia conditions and causes poor prognosis. NLUCAT1 and TRB3 impact hugely on LUAD patient's prognosis in the hypoxia condition [13, 14]. It can be concluded that hypoxia is bound up with the prognosis of LUAD patients.

With deep investigation being carried out, immunotherapy has drawn more and more attention in treating lung cancer. In 2013, Science listed tumor immunotherapy as the top out of 10 breakthroughs in the science field. With the development of immunotherapy (like immune checkpoint inhibitors (ICIs)), methods for tumor therapy are upgraded [15]. The tumor microenvironment comprises surrounding immune and inflammatory cells, tumor cells, tumor-related fibroblasts, and nearby interstitial tissues, capillaries and various cytokines and chemokines. Many immune cells are highly sensitive to hypoxia, with their viabilities and antitumor capabilities lowered in such condition [16]. Lu et al. [17] revealed that proinflammatory cytokines (IL-13 and IL-23) and costimulatory molecules (CD80 and CD60) are downregulated in non-small-cell lung cancer cells, but anti-inflammatory cytokine (IL-10) secreted from the CD1c^+^ DCs is promoted; thereby, blocking the body's antitumor immunity. Ganesan et al. [18] analyzed purified CD8^+^ T cells in untreated, early NSCLC samples by RNA sequencing. The higher the density of tissue-resident memory T cells, the better the performance of LUAD patients' prognoses, but this fact is irrelevant with the density of cytotoxic T lymphocytes.

Meanwhile, immunotherapy was also affected by hypoxia. Presently, the goal of immunotherapy is to induce and enhance cytotoxic T lymphocyte (CTL) effect [19]. In spite of that, hypoxia induces resistance towards CTL. The nuclear translocation of hypoxic cancer cells HIF-1*α*, STAT3 phosphorylation, and VEGF secretion inhibit specific CTL-mediated cell lysis [20]. Additionally, Noman et al.'s study [21] reported hypoxia-induced autophagy as a crucial factor in CTL-mediated innate and adaptive antitumor immunity. Johnson et al. [22] discovered that MHCII can regulate the infiltration of T cells and respond to PD-1 sensitivity. Brooks et al. [23] developed a prognostic classifier for the head and neck cancer through analyzing hypoxia- and immune-related genes. Accordingly, the development of biomarkers based on hypoxia- and immune-related genes is meaningful to clinicians to judge the prognosis of patients.

In recent years, risk signatures based on genes have been widely investigated and applied to predict the prognosis of patients with colon cancer, breast cancer, and hepatocellular carcinoma [24, 25]. In some cancers, their prognostic performance is even better than histopathological diagnosis and tumor staging [24, 25]. However, few studies combined hypoxia and immune characteristics to investigate their relationship with the prognosis of LUAD. This study performed bioinformatics analysis on the prognostic value of hypoxia- and immune-related genes in LUAD, and finally constructed and validated a prognostic risk-assessing signature based on these 7 genes. Finally, tumor mutation burden (TMB) analysis and immune infiltration assessment were used to evaluate the impact of these genes on patients at different risk levels. This signature provides a reference to screening prognostic genes for LUAD patients and to optimizing LUAD immunotherapy.

## 2. Materials and Methods

### 2.1. Data Acquirement

We downloaded the gene expression profiles of 594 LUAD patients from The Cancer Genome Atlas (TCGA, https://portal.gdc.cancer.gov/) database, along with the related clinical information and single nucleotide variants (SNV) mutation data (VarScan2 Annotation, including 561 samples). 243 hypoxia-related genes were downloaded from the Molecular Signatures Database (MSigDB; https://www.gsea-msigdb.org/gsea/msigdb/) [26]. At the same time, the expression data of 1,811 immune-related genes were downloaded from The Immunology Database and Analysis Portal (ImmPort, https://www.immport.org) [27]. GSE31210 dataset downloaded from the Gene Expression database (GEO, https://www.ncbi.nlm.nih.gov/geo/) worked as a validation set. The expression of mRNAs and clinical data of 266 LUAD samples were downloaded as well (raw data were required from GPL570). All datasets used in this study were acquired from public databases, and thus ethnical approval was not needed.

### 2.2. Screening and Enrichment Analysis of the Prognostic Hypoxia- and Immune-Related Genes

To screen prognostic hypoxia- and immune-related genes, 490 tumor samples with survival time exceeding 30 d were selected from TCGA-LUAD dataset as the test set. The test set was intersected with hypoxia- and immune-related datasets. Thereafter, univariate regression analysis (*p* < 0.05) was performed with package “survival” [28] (https://cran.r-project.org/web/packages/survival/index.html) on the dataset required. All the gene sets were aggregated to obtain hypoxia- and immune-related DEGs. To investigate the molecular mechanism of the above-acquired genes, GO (Gene ontology) and KEGG (Kyoto Encyclopedia of Genes and Genomes) were adopted for enrichment analyses (*q* value < 0.05) using “clusterprofile” package (CRAN-Package shadowtext (http://r-project.org)). The results were visualized.

### 2.3. Construction and Evaluation of a Prognostic Signature

LASSO regression was performed using “glmnet” package [29] to reduce the overfitting of genes in building the signature. Multivariate Cox regression analysis was performed with “survival” package. The genes whose LASSO regression coefficient is not 0 were analyzed again, and finally a risk-assessing signature was obtained. The riskScore calculation formula was

(1) $$\begin{array}{c} \text{riskScore}=\overset{n}{\underset{i=1}{\sum }}\exp_{i}*\beta_{i} \end{array}$$

In this formula, *n* represented the number of hypoxia- and immune-related prognostic genes and expi_*i*_ represented the expression value of the hypoxia- and immune-related prognostic genes.  *β*_*i*_ was the coefficient in the multivariate Cox regression analysis. Patient's riskScore was calculated accordingly and patients were divided into the high- and low-risk groups with the median riskScore as the critical value.

“FactoMineR” package was adopted to perform Principal Component Analysis (PCA) [30] on characteristic genes. “Survival” and the “timeROC” packages [31] were applied to draw the Kaplan-Meier (K-M) survival curve and receiver operating characteristic (ROC) curve. The overall survival (OS) of the two groups of patients was compared and analyzed. Then the AUC value of 1-year, 3-year, and 5-year OS was calculated based on the ROC curve to evaluate the predictive ability of the signature. GSE31210 data set was used for verification. Finally, in order to explore whether the riskScore has the value of independently predicting the prognosis of LUAD patients, the riskScore was combined with clinical information to conduct the univariate and multivariate Cox regression analyses.

### 2.4. Enrichment Analysis

To investigate reasons for riskScore divergence, gene set enrichment analysis (GSEA) was carried out on the high- and low-risk groups. We introduced differentially expressed genes (DEGs) to the GSEA software and calculated the Enrichment Score (ES) by comparing the DEGs with gene sets in KEGG pathways. In the line graph for ES, ES > 0 indicated an upregulation of the pathway in the high-risk group. Gene sets with FDR < 0.25 were considered statistically significant.

### 2.5. TMB Analysis

To discuss the way gene mutation frequency and patient's riskScore interplayed, “GenVisR” package [32] was used to calculate the TMB values of the high- and low- risk groups. These values were then subject to the Wilcoxon test using “GenVisR” package. The mutation landscape waterfall chart of different risk groups was drawn.

### 2.6. Immune Infiltration Evaluation

The immune infiltration level and patient's riskScore were evaluated; hereby, “estimate” package [33] and “GSVA” package [34] were utilized to assess the matrix score and immune score of the LUAD samples in the test set. And each LUAD tumor sample was evaluated through single-sample GSEA (ssGSEA).

## 3. Results

### 3.1. Obtaining Prognostic-Related Genes from Hypoxia- and Immune-Related Genes

**Figure 1:**
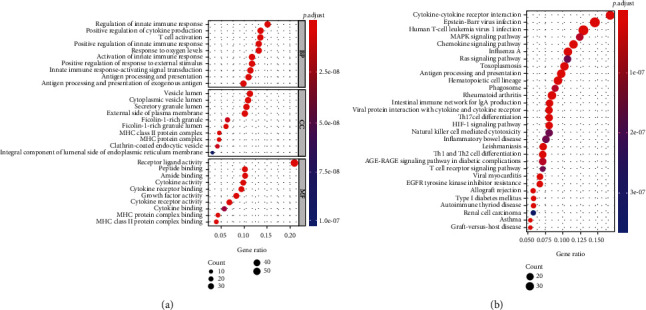
The results of functional enrichment analyses. (a, b) GO and KEGG enrichment analyses.

First, the 243 hypoxia-related genes from the MSigDB and the 1,811 immune-related genes from the ImmPort database were intersected with the genes with mRNA profiles of LUAD patients in TCGA. Then, the univariate Cox regression analysis was adopted to screen 65 prognostic-related hypoxia genes and 213 prognostic-related immune genes. After merging the prognostic-related hypoxia genes and prognostic-related immune genes, totally, 268 prognostic-related genes were obtained (Table S1). The GO enrichment analysis revealed gene enrichment in regulation of innate immune response, positive regulation of cytokine production, and T cell activation (Figure 1(a)). KEGG results suggested that most of these genes were enriched in cytokine-cytokine receptor interaction, MAPK signaling pathway, chemokine signaling pathway, Ras signaling pathway, and antigen processing and presentation (Figure 1(b)). Integrative analyses implicated that the 268 DEGs were mainly associated with the body's innate immune regulatory response.

### 3.2. The Construction of Risk-Assessing Hypoxia- and Immune-Related Prognostic Signature

**Figure 2:**
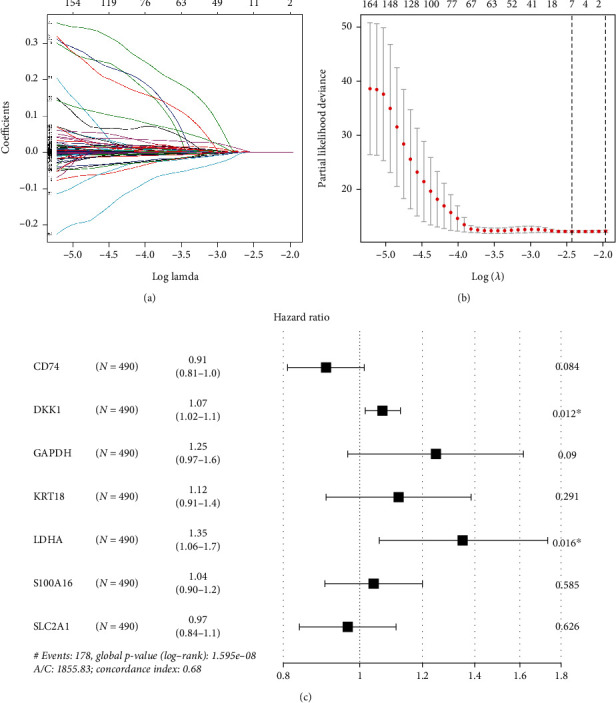
Construction of hypoxia- and immune-related signature. (a) The changing trajectory of coefficients of 268 hypoxia- and immune-related genes with the penalty function *λ* in the LASSO analysis. (b) The selection interval of the best penalty function *λ*. (c) Forest plot manifesting the multivariate Cox regression analysis (∗*p* < 0.05).

The feature genes were screened from 268 DEGs by the LASSO Cox regression analysis. The genes in the range marked within the dotted line were the best range for the identification of signature. Seven important genes were obtained when the LASSO regression coefficient was not 0 (Figures 2(a) and 2(b)). Multivariate Cox regression analysis was performed on these 7 genes and the final 7 hypoxia- and immune-related genes were CD74, DKK1, GAPDH, KRT18, LDHA, S100A16, and SLC2A1. The hazard ratio (HR) values of risk factors (DKK1, GAPDH, KRT18, LDHA, and S100A16) were greater than 1, and the HR values of protective factors (CD74 and SLC2A1) were less than 1 (Figure 2(c)). The final LUAD signature was riskScore = −0.09897∗CD74 + 0.06688∗DKK1 + 0.2221∗GAPDH + 0.1131∗KRT18 + 0.3026∗LDHA + 0.03997∗S100A16 − 0.03507∗SLC2A1.

### 3.3. Validation of the Prediction Ability of the 7-Gene Based Risk-Assessing Signature

**Figure 3:**
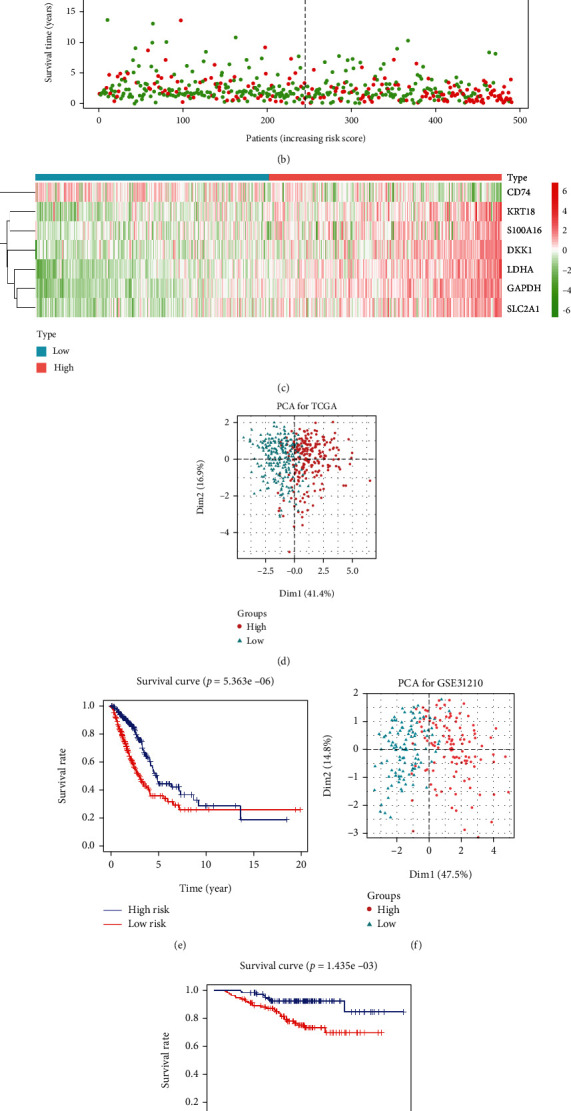
The evaluation of the prediction performance of the 7-gene based risk-assessing signature. (a) RiskScore distribution map of LUAD samples, where green represents low risk, and red represents high risk. (b) Scatter plot manifests the distribution of survival status in the high- and low-risk groups, where green represents survival samples, and red represents dead samples. (c) Heat map showing the expression of 7 characteristic genes, where green means low expression, and red means high expression. (d) PCA cluster map of test set and (f) validation set in the high- (red) and low-risk (cyan) groups. (e) The test set and (g) the K-M survival curve of the high- (red) and low-risk (blue) groups in the validation set.

The riskScore of the LUAD samples was calculated according to the established risk-assessing signature, and samples were divided into high- and low-risk groups according to the median value of riskScore (Figure 3(a)). A scatter plot of patient's survival time was drawn based on the grouping. The results revealed that as riskScore increased, patient's survival time was shortened and their mortality increased (Figure 3(b)). At the same time, a heat map indicated that with the increase of the riskScore, the expression of DKK1, GAPDH, KRT18, LDHA, S100A16, and SLC2A1 increased, while the expression of CD74 decreased (Figure 3(c)). The PCA results suggested that patients in different groups could be clustered clearly according to the defaults (Figure 3(d)). K-M survival curve unveiled that the prognosis of patients with low riskScore was often better than those with high one (Figure 3(e)). PCA and survival analysis were performed on both groups with GSE31210 as the validation set. The results of the validation set were similar to the test set (Figures 3(f) and 3(g)).

**Figure 4:**
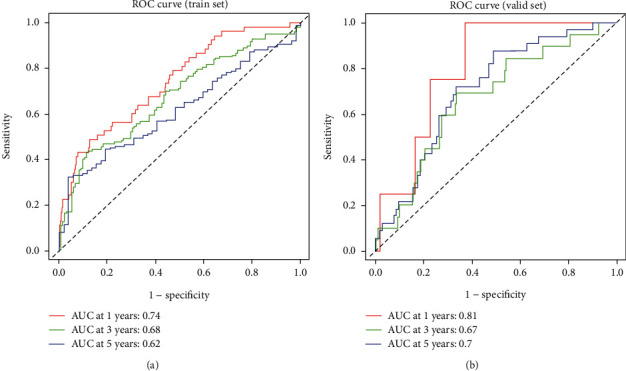
The evaluation of 7-gene based risk-assessing signature. (a, b) ROC curves of the test and validation sets.

Based on ROC curves, the AUC values of 1-, 3-, and 5-year OS were 0.74, 0.68, and 0.62, respectively (Figure 4(a)). The AUC values of 1-, 3-, and 5-year OS in the validation set was 0.81, 0.67, and 0.7, respectively (Figure 4(b)). The above results revealed that the signature-based riskScore predicted the prognosis of LUAD patients robustly.

**Figure 5:**
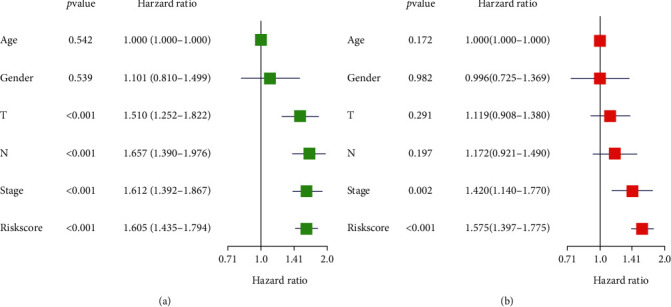
The Cox regression analysis of factors relating to patient's prognosis. (a, b) The results of the univariate and multivariate Cox regression analyses.

In order to explore whether the riskScore could independently assess the prognosis of LUAD patients, we combined the clinical data of the patients, including age, sex, and tumor stage, to perform the univariate Cox regression analysis. The results revealed that the riskScore and the traditional clinical prognostic factors T stage, N stage, and clinical stage were prominently correlated with patients' OS (Figure 5(a)). Multivariate Cox regression analysis of the above results suggested that the riskScore and clinical stage were notably correlated with patients' OS (Figure 5(b)). The riskScore according to the signature were independent enough to be the prognostic factor of LUAD.

### 3.4. GSEA of the High- and Low-Risk Groups

**Figure 6:**
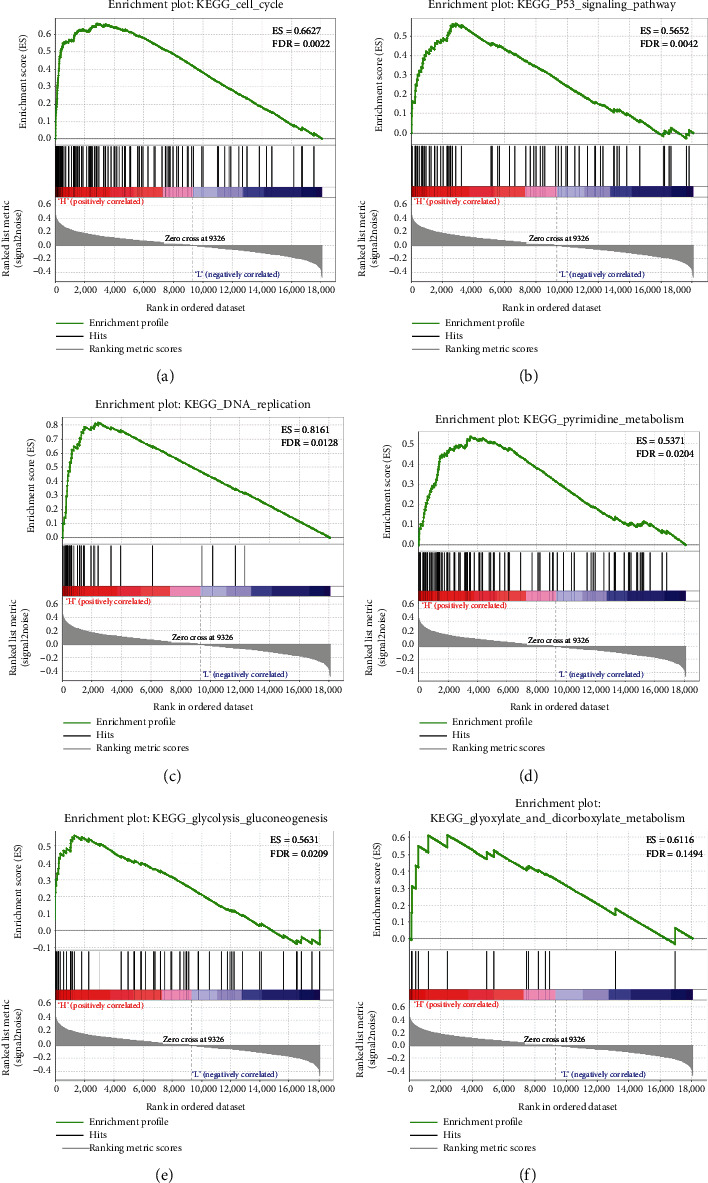
The 7 genes in the high- and low-risk groups were enriched in (a) cell cycle; (b) p53 signaling pathway; (c) DNA replication; (d) pyrimidine metabolism; (e) glycolysis and gluconeogenesis; and (f) glyoxylate and dicarboxylate metabolism.

GSEA software was utilized to explore the reason for riskScore divergency. The results suggested that the expression of these 7 genes in the high-risk group was remarkably enriched in p53 signaling pathway, cell cycle, DNA replication, pyrimidine metabolism, glycolysis and gluconeogenesis, and glyoxylate and dicarboxylate metabolism (Figure 6). These pathways were all related to cancer progression, indicating a reliable performance of the 7-gene prognostic risk model.

### 3.5. Analysis of Mutation Characteristics of Genes in LUAD

**Figure 7:**
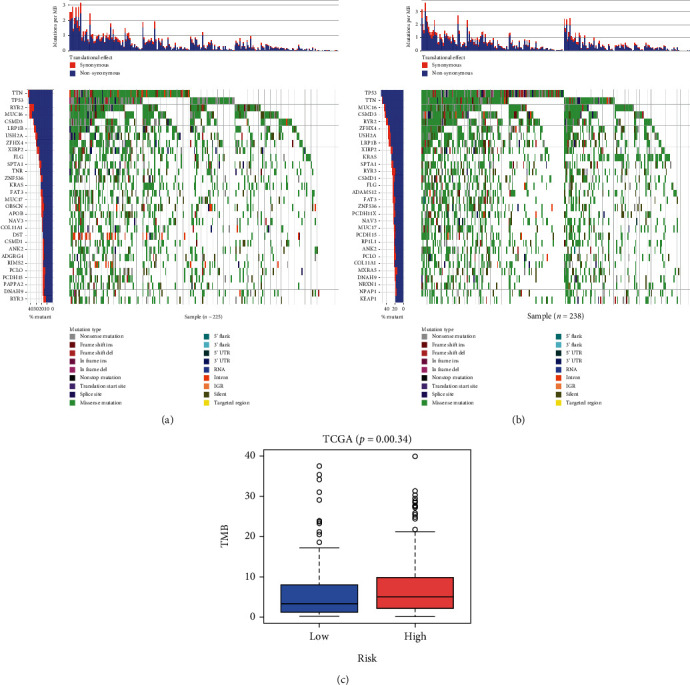
Mutation characteristics of genes in LUAD patients. (a, b) Waterfall chart of top30 genes in the low- and high-risk groups, with the horizontal axis represents different samples, while the vertical axis indicates the gene. The filled means that the gene is mutated. Different colors represent different mutation types. (c) Differences in TMB in the high- and low-risk groups.

With the increasing application of immunotherapy in treating LUAD, improving the efficiency of immunotherapy has drawn considerable attention. At present, studies have confirmed that TMB works as a biomarker to verify the effectiveness of immunotherapy. To explore the relationship between riskScore and TMB in LUAD, we analyzed the genes in different risk groups and found that there were differences in TMB between the high- and low-risk groups (Figures 7(a) and 7(b)). Subsequently, the Wilcoxon test was performed on the TMB values, and the results indicated a remarkable high TMB in the high-risk group (Figure 7(c)). Hence, we speculated that changes in gene hypoxia and immune characteristics might affect the frequency of gene mutations.

### 3.6. Evaluation of Immune Infiltration

**Figure 8:**
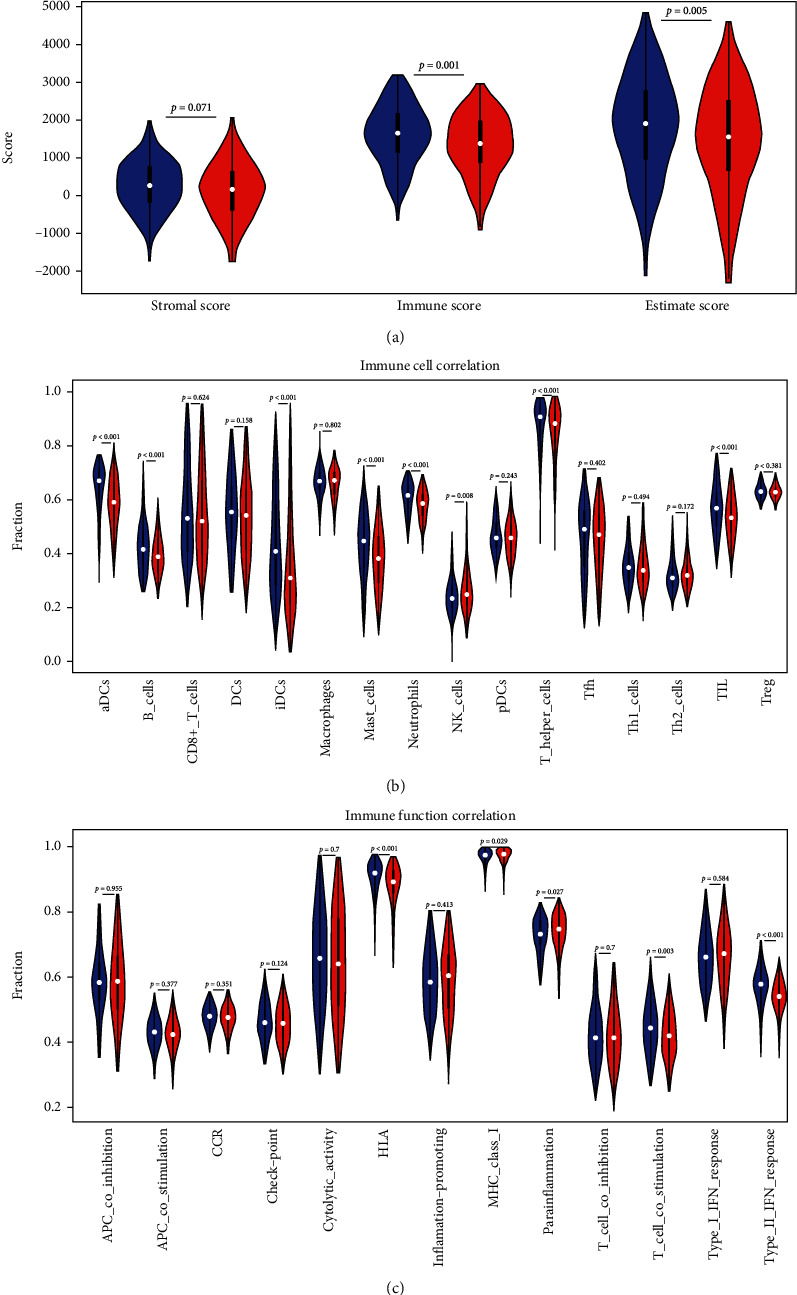
Immune infiltration assessment results. (a) Differential analysis of stromal scores, immune scores, and estimate scores. (b) Differential analysis of immune cell components. (c) Differential analysis of immune functions. Blue represents the low-risk group, and red represents the high-risk group.

The “estimate” package was utilized to score the stromal cell components and immune cell components of LUAD tumor samples. Tumor purity was calculated and differentially analyzed. The results indicated that the immune scores and estimate scores of the high-risk group were significantly lower than those of the low-risk group (Figure 8(a)). The results of ssGSEA analysis revealed that compared with the high-risk group, the low-risk group had higher levels of immune infiltration in aDCs, iDCs, mast cells, B cells, neutrophils, TIL, and T helper cells (Figure 8(b)). While the immune activities in HLA, type II IFN response, and T cell costimulation were higher in the low-risk group (Figure 8(c)). On the above, we concluded relatively low immune scores of patients in the high-risk group, which may impact the immunotherapy of LUAD.

## 4. Discussion

With the increasing development of high-throughput technology and research in LUAD, the treatment of LUAD has improved, especially in immunotherapy. However, the therapeutic effect and patient's prognosis are still not optimistic. In recent years, biomarkers have been proven to guide patients' diagnosis, treatment, and prognosis prediction. Based on the TCGA database, this study analyzed the hypoxia and immune characteristics of LUAD and combined the two to build a prognostic-assessment signature based on 7 genes for LUAD patients.

The seven genes for constructing a risk-assessing signature for prognosis were as follows: CD74, DKK1, GAPDH, KRT18, LDHA, S100A16, and SLC2A1, among which the four genes CD74, DKK1, GAPDH, and S100A16 were related to immunity. S10016A is a member of the S100A family. Studies have found that S100A16 can inhibit the immune infiltration of CD8^+^ T cells through the focal adhesion-Ras-stimulated signaling pathway in pancreatic cancer [35]. The study of Ou et al. [36] found that S100A16 inhibits activities of CRC cells through the JNK/p38 MAPK signaling pathway and subsequent EMT. DDK1 encodes secreted proteins and regulates protein-protein interactions [37]. Especially in myeloma cells, DKK1 was secreted to inhibit function of osteocytes [37]. Some scholars found that multiple myeloma cells can phosphorylate cAMP-responsive element-binding protein (CREB) through p38 kinase under hypoxic conditions and drive CREB into the nucleus to activate DKK1 transcription [38]. CD74 is a type II transmembrane glycoprotein and becomes the proinflammatory cytokine macrophage migration inhibitor in an inflammatory environment [39]. Studies have found that in pancreatic cancer, CD74 expression is related to perineural infiltration and the poor prognosis of patients after surgical resection [40]. Although GAPDH can be stably expressed as a housekeeping protein most of the time, there have been studies taking GAPDH as a prognostic gene and a potential therapeutic target for certain cancers [41, 42]. Thus, the immune-related genes tested in this study were closely related to the prognosis and immunotherapy.

KRT18, LDHA, and SLC2A1 were three characteristic genes relating to hypoxia. Keratin 18 is one of cytoskeletal proteins and functions in various cancers. Works found that KRT18 facilitated the progression of gastric cancer and is relating to the prognosis of GC patients [43]. LDHA-encoded proteins are involved in the last step of anaerobic glycolysis and catalyze the transformation of L-lactic acid and NAD. Studies found that the accumulation of LDHA-related lactic acid in melanoma can repress the function and activity of T cells and NK cells, leading to immune escape of tumors [44]. SLC2A1 encodes the unidirectional protein GLUT-1 and involves in glucose transport. Studies have uncovered that GLUT-1 is related to the cell proliferation of pancreatic cancer cells and is an important regulator in the prognosis of patients with pancreatic cancer [45, 46]. On the above, the selected 7 genes in this study were associated with hypoxia, the tumor microenvironment, and patient's prognosis.

With the development of immunotherapy, people have paid more and more attention to what characteristics of patients could benefit from immunotherapy. Current studies have confirmed that TMB can be a biomarker for immunotherapy, and immunotherapy is quite effective in patients with high TMB [47]. The evaluation of TMB and tumor immune infiltration is important in evaluating whether the patient receives immunotherapy and the efficiency of immunotherapy. Zhang et al. [48] analyzed the head and neck squamous cell carcinoma and found that patient's immunotherapy is related to the patient's TMB and tumor immune infiltration, and the ICI score constructed based on tumor immune infiltration is a predictor of immunotherapy free from TMB. The study by Kang et al. [49] confirmed that in melanoma, the relationship between TMB and immune infiltration, especially the abundance of macrophages and Tregs, could lead the prediction signature of immunotherapy response. This study combined the riskScore with the TMB and immune infiltration assessment of LUAD patients and discovered high TMB scores and low immune scores in the high-risk group. Combined with existing studies, this study inferred that patients with a high riskScore may reflect a relatively high TMB value and a relatively low immune score, which is a proper condition for immunotherapy, thereby producing a better prognosis.

However, this study also has certain limitations. First, genetic data used in this study were based only on public databases. The risk-assessing signature for the prognosis of LUAD patients was constructed on the basis of the hypoxia- or immune-related DEGs in LUAD patients. Secondly, although TMB and immune infiltration assessment analysis were carried out on LUAD patients, subsequent clinical trials were required to verify the results of bioinformatics analysis.

In summary, we generated a risk-assessing signature for the prognosis of LUAD patients based on 7 hypoxia- and immune-related genes via the LASSO and the Cox regression analysis. It predicted patient's prognosis robustly, and the riskScore could be regarded as an important prognostic assessment factor independent of clinical characteristics. At the same time, the hypoxia- and immune-related genes of the constructed signature were likely to be potential targets of LUAD treatment, which provided reference to determining the prognosis and making clinical treatment plans for LUAD patients.

## Data Availability

All data generated or analyzed during this study are included in this article.

## Supplementary Materials

Supplementary Materials Table 1: Details of 268 prognostic-related DEGs.
