# Supplementary material for: A Risk-Assessing Signature Based on Hypoxia- and Immune-Related Genes for Prognosis of Lung Adenocarcinoma Patients
Source: Comput Math Methods Med. 2022 Sep 28;2022:7165851. doi: 10.1155/2022/7165851 (PMC9534655; doi:10.1155/2022/7165851)
Supplement: Supplementary Materials — Table 1: Details of 268 prognostic-related DEGs. [file 7165851.f1.pdf]

gene	HR	HR.95L	HR.95H	pvalue
LDHA	1.001256414	1.000906138	1.001606813	2.02E-12
GAPDH	1.000192734	1.000122019	1.000263455	9.18E-08
SLC2A1	1.001782801	1.001121227	1.002444813	1.26E-07
PKM	1.001275939	1.000742192	1.00180997	2.77E-06
KRT18	1.000232719	1.000133077	1.000332372	4.70E-06
STC2	1.016576417	1.009343812	1.023860849	6.39E-06
ITGA5	1.003066545	1.00172627	1.004408614	7.18E-06
ANGPTL4	1.002158292	1.001194931	1.003122579	1.11E-05
FKBP4	1.002647766	1.001366831	1.003930341	5.03E-05
TH	1.24158424	1.11729656	1.379697638	5.80E-05
PGAM1	1.010243721	1.005187472	1.015325403	6.86E-05
GPI	1.002772508	1.001400292	1.004146604	7.41E-05
TPI1	1.000666311	1.000334482	1.00099825	8.28E-05
VEGFC	1.007570325	1.003782698	1.011372243	8.68E-05
ENO1	1.000357239	1.000178555	1.000535955	8.90E-05
ANXA2	1.001112657	1.000531958	1.001693694	0.000172363
ADM	1.005010348	1.002356208	1.007671516	0.000211999
DDIT4	1.001035937	1.000474267	1.001597923	0.000299437
CD99	1.00242723	1.00109716	1.003759067	0.000345337
NFKB2	1.007621286	1.003413565	1.011846653	0.000376463
BIK	1.009161277	1.004075723	1.014272588	0.000403291
SERPINE1	1.001205435	1.000532818	1.001878504	0.00044225
PAICS	1.004544667	1.002006689	1.007089072	0.000442817
PLOD2	1.002338942	1.001001669	1.003678002	0.000604096
SLC16A1	1.003820933	1.001542662	1.006104387	0.001003201
ALDOA	1.000436191	1.000175635	1.000696815	0.001032847
HSPD1	1.001198672	1.000474974	1.001922893	0.001165916
BCL2L1	1.002990678	1.001143308	1.004841458	0.00149957
XRCC6	1.001696548	1.000613341	1.002780928	0.002135658
IGFBP3	1.000627895	1.000225198	1.001030753	0.002240344
COL4A5	1.020027725	1.007004896	1.033218967	0.002488584
HLA-DQB1	0.998807624	0.998018528	0.999597344	0.003089423
HERPUD1	0.995534677	0.992554148	0.998524156	0.003440191
TPBG	1.011797717	1.003736245	1.019923935	0.004057046
MIF	1.002013046	1.000629307	1.003398699	0.004341398
IGFBP1	1.001835252	1.000568719	1.003103388	0.004499119
GBE1	1.011384731	1.003389091	1.019444085	0.005182811
TGFBI	1.000614501	1.000183201	1.001045987	0.005226278
TXN	1.000354426	1.000099977	1.00060894	0.006329566
KRT19	1.00017812	1.000048695	1.000307561	0.006987272
PPAT	1.013689127	1.003534532	1.023946474	0.00812504
P4HA1	1.001942481	1.000502313	1.003384722	0.008187494
CDK1	1.004958488	1.001255519	1.008675151	0.008635664
ERRFI1	1.000610482	1.000152196	1.001068978	0.00902606
SLC20A1	1.004627008	1.001142165	1.008123981	0.009218808
SLC3A2	1.002675634	1.00065648	1.004698863	0.009375436
LOX	1.004714183	1.001150736	1.008290315	0.009476144
SLC6A8	1.005169291	1.001194187	1.009160177	0.010763344
PNP	1.005043521	1.001107555	1.008994962	0.01197519
SIAH2	1.007931563	1.001718016	1.014183653	0.01227875

NT5E	1.001884226	1.000385123	1.003385576	0.013741263
UMPS	1.024923944	1.004776504	1.045475374	0.015082605
RELA	1.008202773	1.001471235	1.014979557	0.01684447
PDGFB	1.009246065	1.001566748	1.016984262	0.018192114
AK2	0.994824744	0.990550097	0.999117839	0.018193238
CCT6A	1.000371717	1.000061997	1.000681533	0.018654575
PGK1	1.000574258	1.000056156	1.001092629	0.029820893
HK2	1.002860305	1.000222842	1.005504723	0.03351994
P4HA2	1.006826252	1.000466072	1.013226864	0.03537153
HGF	0.964793862	0.932684883	0.998008238	0.037948097
CDKN1A	1.001775583	1.000074273	1.003479787	0.040795262
PSMA3	1.003011823	1.000112787	1.005919261	0.041717093
PGM1	1.003612864	1.000107858	1.007130154	0.043343365
HSPH1	1.005265903	1.000135493	1.010422629	0.044234436
F3	1.000586146	1.000010623	1.001162	0.045917276
CXCL5	1.002944841	1.00190101	1.00398976	3.12E-08
S100A16	1.00088937	1.000536489	1.001242377	7.77E-07
DKK1	1.002589257	1.001451035	1.003728773	8.13E-06
IL11	1.041758872	1.023143346	1.060713098	8.71E-06
FGF12	1.041026835	1.022738953	1.059641727	8.73E-06
PSMD11	1.008965971	1.004875125	1.01307347	1.66E-05
PSMD2	1.003063283	1.00166129	1.004467239	1.82E-05
S100A10	1.000408196	1.000219462	1.000596966	2.24E-05
OXTR	1.025593997	1.01361726	1.037712249	2.48E-05
STC1	1.002750074	1.001452173	1.004049656	3.24E-05
TNFRSF11A	1.04752233	1.024043492	1.071539481	5.96E-05
RAET1E	1.058593257	1.029300558	1.088719593	6.98E-05
F2RL1	1.006023103	1.003025826	1.009029337	8.00E-05
SHC1	1.002342521	1.001168414	1.003518006	9.13E-05
JAG1	1.008479444	1.004127216	1.012850535	0.000129999
HSPA4	1.00746943	1.003564287	1.011389769	0.00017298
NRG4	1.114748556	1.052567125	1.180603415	0.000207737
SEMA4B	1.002219716	1.001044929	1.003395883	0.000211215
PROCR	1.003216254	1.001474573	1.004960964	0.000292323
TNFRSF1A	1.003859922	1.001731425	1.005992941	0.000374585
NEDD4	1.034234829	1.015054637	1.053777445	0.000424331
ADIPOR2	1.006658862	1.002950277	1.010381161	0.000424524
INSL4	1.004537526	1.001976847	1.007104749	0.000508027
SEMA3C	1.005686277	1.002460283	1.008922652	0.000542262
SEMA7A	1.005204645	1.002240658	1.008177398	0.000570066
FURIN	1.000274807	1.000116927	1.000432712	0.000645531
HLA-DMA	0.998216892	0.99717926	0.999255604	0.000770097
FGF2	1.06183451	1.024823901	1.100181725	0.000917645
LANCL1	1.008411038	1.003419448	1.013427459	0.000938787
HLA-DRA	0.999911723	0.999859399	0.999964049	0.000944673
CD74	0.999914204	0.999862767	0.999965642	0.00107914
KRAS	1.003238917	1.001276256	1.005205425	0.00120993
PSMC5	1.007468513	1.00292552	1.012032084	0.00125177
KLRC2	1.064142791	1.024503192	1.105316106	0.001328222
HLA-DMB	0.994981786	0.99192824	0.998044733	0.001336679
S100A11	1.00012738	1.000049227	1.00020554	0.001400375

BTK	0.966388275	0.946250604	0.986954506	0.001461899
PAK2	1.006983507	1.002660292	1.011325363	0.001523168
HNF4A	1.022653919	1.008568684	1.036935862	0.001546919
GHRHR	1.34407929	1.118104366	1.615724964	0.001640472
HLA-DPB1	0.99934872	0.998943234	0.99975437	0.001653063
HLA-DPA1	0.998985105	0.9983519	0.999618711	0.001696258
BIRC5	1.005667483	1.002098412	1.009249265	0.001835877
IL1R2	1.007483491	1.002762298	1.012226913	0.001864598
HLA-DRB5	0.999713054	0.999532149	0.999893991	0.001882856
RAC1	1.001291357	1.000476725	1.002106653	0.001885615
BMP7	1.008089117	1.002979082	1.013225187	0.001888625
PPIA	1.00178008	1.000655715	1.002905709	0.001909196
RARG	1.009733796	1.003560797	1.015944766	0.001961316
SLC10A2	1.009868656	1.003594192	1.016182348	0.00201363
CRABP1	1.000898409	1.000328097	1.001469047	0.00201493
XCR1	0.818137731	0.718964844	0.93099037	0.002330193
TAP2	1.010280186	1.003640681	1.016963614	0.002364254
LCN15	1.017956677	1.006261444	1.029787838	0.002538774
SEMA4G	1.014124098	1.004876801	1.023456493	0.002692022
S100P	1.000141019	1.000048788	1.000233258	0.002728228
BDNF	1.060093826	1.020277834	1.101463623	0.002810376
LTBR	1.004017295	1.001379231	1.006662309	0.002819805
ACTG1	1.000113283	1.000038201	1.000188371	0.003104166
CD40LG	0.927384682	0.882180162	0.974905563	0.003108941
FCGRT	0.996713573	0.994525204	0.998906758	0.003331767
SOD1	1.001329362	1.000435752	1.00222377	0.003541674
CBLC	1.004057401	1.001313554	1.006808767	0.003729795
INHA	1.002287322	1.000729671	1.003847397	0.003987541
HLA-DOB	0.982315054	0.970383495	0.99439332	0.004213829
HLA-DRB1	0.999865275	0.999772776	0.999957783	0.004312274
CX3CR1	0.954925855	0.925147027	0.985663209	0.004326274
OAS1	1.002268062	1.000700288	1.003838293	0.004562155
HLA-DQA1	0.998073274	0.996740557	0.999407774	0.004670525
FGF8	1.170140581	1.048800249	1.305519313	0.004908394
UCN2	1.039471936	1.011596	1.068116032	0.005250686
ADRB2	0.953085058	0.921287093	0.985980521	0.005512203
CAT	0.996060677	0.993279152	0.998849991	0.005667083
IL20RB	1.00560462	1.001604132	1.009621086	0.005994568
VIPR1	0.964629235	0.940001089	0.989902642	0.006351448
PPARG	1.009616056	1.00269322	1.016586689	0.006408451
CACYBP	1.005825525	1.001624554	1.010044116	0.006526126
MAP2K1	1.007525056	1.002051615	1.013028394	0.006988774
HSP90AA1	1.000465858	1.000125426	1.000806405	0.007312931
NTS	1.000093528	1.000025073	1.000161987	0.007409125
CCR6	0.500681807	0.300946225	0.832980283	0.007731127
BMP1	1.012224622	1.003141177	1.021390318	0.008244777
CD1D	0.947953729	0.91109605	0.986302457	0.008251321
MC1R	1.05732986	1.013766049	1.102765706	0.00940815
HSPA2	1.00792399	1.001933698	1.013950096	0.009454869
NRAS	1.004810181	1.001167459	1.008466158	0.009608099
TPM2	1.002167637	1.000524508	1.003813463	0.009701574

IL16	0.96075099	0.931927585	0.990465868	0.009984007
TLR2	0.993838694	0.989156733	0.998542817	0.010310931
HLA-DOA	0.9961173	0.993159555	0.999083854	0.010345162
CMTM7	0.985767866	0.97491165	0.996744972	0.011180636
PIK3CG	0.957391345	0.925666897	0.990203052	0.011322222
IGF1R	1.008239367	1.001843219	1.014676351	0.011500796
CIITA	0.981001585	0.966400223	0.99582356	0.012177331
PLXNA4	1.061173747	1.013023987	1.1116121	0.012205965
ITGAL	0.987939282	0.978592609	0.997375227	0.012354055
QRFP	1.226930487	1.045158512	1.440315896	0.012424653
CRP	1.016497123	1.003508965	1.029653384	0.012637169
GMFG	0.994178462	0.989595085	0.998783067	0.013269783
PAK1	1.006618189	1.001361898	1.011902071	0.013531472
RAET1G	1.028179205	1.005739711	1.051119357	0.013574903
SCGB3A1	0.999902615	0.99982526	0.999979977	0.013616233
PTPN6	0.990141298	0.982354017	0.99799031	0.013919984
RELB	1.008947823	1.001786645	1.016160192	0.014240462
LGR4	1.003954878	1.000787251	1.007132531	0.014363701
PTGDS	0.997816979	0.996069085	0.99956794	0.014562977
TLR7	0.951294562	0.913865638	0.990256452	0.014766547
CCL3L3	0.962177019	0.932739829	0.992543244	0.015012281
PLSCR1	1.003536288	1.000673846	1.006406919	0.015428008
IL1RAP	1.021889676	1.004046858	1.040049577	0.015981398
TAP1	1.001176527	1.000207848	1.002146144	0.017277042
SFTPA2	0.999969017	0.999943474	0.99999456	0.017437083
TNFSF12	0.991236087	0.984030363	0.998494575	0.018045658
IL2	0.493081387	0.273637419	0.888508799	0.018602102
IL33	0.992857086	0.986944299	0.998805297	0.018661917
EGFR	1.00148392	1.000237415	1.002731977	0.01961955
PTPRC	0.993810434	0.988625843	0.999022213	0.01999001
TNFRSF17	0.986671018	0.975521083	0.997948393	0.020659891
VAV2	1.006928932	1.001045754	1.012846685	0.020912554
ARRB1	0.991309076	0.98395458	0.998718543	0.021592452
TNFSF13	0.989370641	0.980372119	0.998451757	0.021886059
SFTPA1	0.999963452	0.999932188	0.999994718	0.021960274
CSF2RB	0.987057233	0.976107616	0.998129678	0.022085923
DDX58	1.005711695	1.000811235	1.01063615	0.022292481
IL22RA1	1.00694326	1.000975171	1.012946932	0.022529059
RARB	1.026576322	1.003449755	1.050235889	0.024058445
SEMA4A	0.988797817	0.979156004	0.998534573	0.024240744
PRLR	1.021307753	1.002732427	1.040227182	0.024364404
A2M	0.999634033	0.999315411	0.999952757	0.024421688
FGR	0.989039681	0.979546081	0.998625291	0.025122982
NR2F2	1.005021365	1.000623533	1.009438526	0.025185304
PRKCA	1.010680201	1.001259884	1.020189149	0.02618286
PIK3CD	0.984609787	0.971234118	0.998169663	0.026250756
NOD1	0.980113781	0.962862539	0.997674107	0.026624903
CTSG	0.959474749	0.924984436	0.995251118	0.026772866
ABCC4	0.988831921	0.97902658	0.998735466	0.027187059
TNFSF14	1.046363947	1.005051488	1.089374548	0.027445118
AGER	0.99892292	0.997961447	0.99988532	0.0282794

EPGN	1.014731307	1.001553269	1.028082736	0.028330585
IL5RA	0.88811805	0.798721174	0.987520671	0.028382506
HSPA1A	1.001154118	1.000118848	1.00219046	0.028881805
S100A2	1.000299475	1.000030323	1.000568698	0.029197433
CTF1	0.988959883	0.979125599	0.998892943	0.029465852
PPARD	1.005446818	1.000540354	1.010377342	0.029525053
NOX1	1.023150095	1.00223532	1.044501323	0.029866622
CD1E	0.979488034	0.961307263	0.99801265	0.030153561
ANGPTL5	0.532063328	0.300316859	0.942642334	0.030588199
CD81	0.99845445	0.997052816	0.999858055	0.030926338
LIFR	0.988952045	0.979004523	0.999000643	0.031255226
PML	1.009150802	1.000765788	1.017606071	0.032372415
IL6R	0.992248326	0.985163734	0.999383866	0.033291963
TNFRSF10C	0.981358281	0.964479512	0.998532435	0.033513226
CD1B	0.957582301	0.920039364	0.996657206	0.033665806
SEMA3A	1.007631909	1.000586557	1.014726868	0.033690077
PAK4	1.007328565	1.000557834	1.014145114	0.033834845
CCL14	0.90637875	0.827682813	0.992557082	0.033907027
GNAI1	1.007904137	1.000594565	1.015267108	0.034004548
NFATC1	0.956681065	0.918150114	0.996829001	0.03473897
AVPR1B	1.257419254	1.015610334	1.556800997	0.035543238
ARG2	0.982554534	0.966517874	0.998857278	0.036069476
IL15RA	1.011616851	1.000743095	1.022608759	0.036199353
GDF10	0.973148863	0.948621754	0.998310133	0.036633777
RFXANK	0.992815789	0.986106408	0.99957082	0.03715634
CCL17	0.994707631	0.989751469	0.999688612	0.03732717
CMTM5	0.146928914	0.024009569	0.899145927	0.037987747
CD1C	0.990209144	0.981027455	0.999476768	0.038445122
TRIM22	0.994639641	0.989589468	0.999715586	0.038500163
UCN3	1.000378064	1.000019146	1.00073711	0.038967029
SHC3	0.951965766	0.908477631	0.997535645	0.03907573
RBP2	1.011611614	1.000573687	1.022771307	0.039167602
MAP3K8	0.986007614	0.972820448	0.999373541	0.040249128
CCL23	0.970879537	0.943736804	0.998802919	0.041076365
CHP2	1.032017437	1.001264762	1.063714645	0.041165958
FLT3	0.84465268	0.717977598	0.993677452	0.041703144
LGR5	1.005766459	1.000211612	1.011352157	0.041866579
STAT1	1.000732295	1.000026478	1.001438611	0.042000289
OSTN	1.25595754	1.00813225	1.564704771	0.042134767
PLCG2	0.967683004	0.937483286	0.998855564	0.042280287
GMFB	1.007654714	1.000242413	1.015121944	0.042938488
IL17RD	1.021838811	1.00062888	1.043498321	0.043517411
NR3C2	0.977897649	0.956901159	0.999354848	0.043566233
TGFB2	1.010431258	1.000283921	1.020681534	0.043895324
TLR8	0.973165851	0.947739342	0.999274517	0.044041933
VIPR2	1.054596248	1.001280221	1.110751239	0.044611465
FGA	1.000069554	1.000001668	1.000137446	0.044630856
CCL21	1.000316572	1.000007206	1.000626033	0.044896079
PSME3	1.003463185	1.000077438	1.006860395	0.044977025
ICOS	0.954248379	0.911488212	0.99901453	0.045272341
KL	0.94193185	0.888346245	0.998749773	0.045303413

NR1H4	1.041092274	1.000701411	1.083113416	0.046076219
CCR4	0.971885391	0.944945255	0.999593583	0.04677784
PLXND1	0.996826931	0.993704931	0.999958741	0.04706174
PSMC1	1.010802235	1.000132105	1.021586201	0.047215633
PRTN3	1.111148769	1.001178981	1.233197669	0.047465397
TYROBP	0.999328522	0.998664526	0.999992961	0.047622528
CD79A	0.998311425	0.99664124	0.999984409	0.047904527
PSMC4	1.001671236	1.000008835	1.0033364	0.048793733
PSMD7	1.003671285	1.000013255	1.007342697	0.049174104
SERPIND1	0.996518426	0.993056388	0.999992534	0.049510504
NMB	1.002904101	1.000004621	1.005811988	0.049635498
SFTPD	0.999806071	0.999612405	0.999999776	0.049735284
PLXNA2	0.989577185	0.979267196	0.99999572	0.049906449
FGFR1	1.002378114	1.000000229	1.004761655	0.049977961
